# Peroral pancreatoscopy–guided laser stricturoplasty enabling laser lithotripsy for an inaccessible pancreatic stone due to a tight stricture

**DOI:** 10.1055/a-2961-1627

**Published:** 2026-10-08

**Authors:** Yusuke Takasaki, Yusuke Yamaguchi, Haruka Hagiwara, Sho Takahashi, Ko Tomishima, Toshio Fujisawa, Hiroyuki Isayama

**Affiliations:** 1 Department of Gastroenterology 73362 Juntendo University School of Medicine Graduate School of MedicineBunkyo-kuTokyoJapan; 2 Division of GastroenterologyDepartment of Medicine, Faculty of Medicine 26683 Chulalongkorn UniversityBangkokBangkokThailand

## Abbreviations



**Video 1** Complete stone clearance by peroral pancreatoscopy–guided laser stricturoplasty followed by laser lithotripsy for pancreatic stones.

POPS: peroral pancreatoscopy
LL: laser lithotripsy
PLS: pancreatoscopy-guided laser stricturoplasty.

Chronic pancreatitis often leads to pancreatic duct strictures, resulting in pancreatic stone formation. In addition to extracorporeal shock wave lithotripsy, peroral pancreatoscopy (POPS)–guided electrohydraulic lithotripsy and laser lithotripsy (LL) are used to treat pancreatic stones. However, tight strictures may prevent POPS from reaching upstream stones. Recently, pancreatoscopy-guided laser stricturoplasty (PLS) has emerged as a potential option for the management of fibrotic strictures. 1​ 2​ 3​ ​4​ We report a case in which PLS restored access to recurrent upstream pancreatic stones, enabling LL and complete stone removal. A 31-year-old man with alcoholic chronic pancreatitis had previously undergone endoscopic pancreatic stone removal. Recurrence of a pancreatic duct stricture and multiple upstream pancreatic stones was detected, and retreatment was planned. Fluoroscopy showed a stricture in the pancreatic body with multiple stones upstream ( Fig. 1 ). After endoscopic papillary balloon dilation using a 6-mm balloon (REN 6 mm; KANEKA, Osaka, Japan), a POPS (SpyGlass DS II; Boston Scientific, Marlborough, Massachusetts, USA) was advanced. Direct visualization revealed a fibrotic stricture with stones beyond it. Because the POPS could not pass the stricture, PLS was performed using a laser (Quanta Litho EVO; EDAP TMS S.A., Vaulx-en-Velin, France) at 0.8 to 1.0 J and 10 Hz. We initially attempted a shaving-off technique, 5​ but due to limitations in probe maneuverability, we decided to make slits from distal to proximal in the lower half of the stricture ( Fig. 2 ). After PLS, the stricture was dilated with a 6-mm balloon. Repeat POPS confirmed adequate expansion, allowing easy passage of the scope. LL was then performed at 1.5 J and 8 Hz, followed by basket extraction with a SpyBasket (Boston Scientific; Fig. 3 ). Final pancreatography and computed tomography revealed marked improvement of the stricture and complete stone clearance ( Fig. 4 , Video 1 ). PLS followed by LL may be an effective endoscopic treatment strategy for pancreatic duct strictures associated with pancreatic stones.

**Fig. 1:**
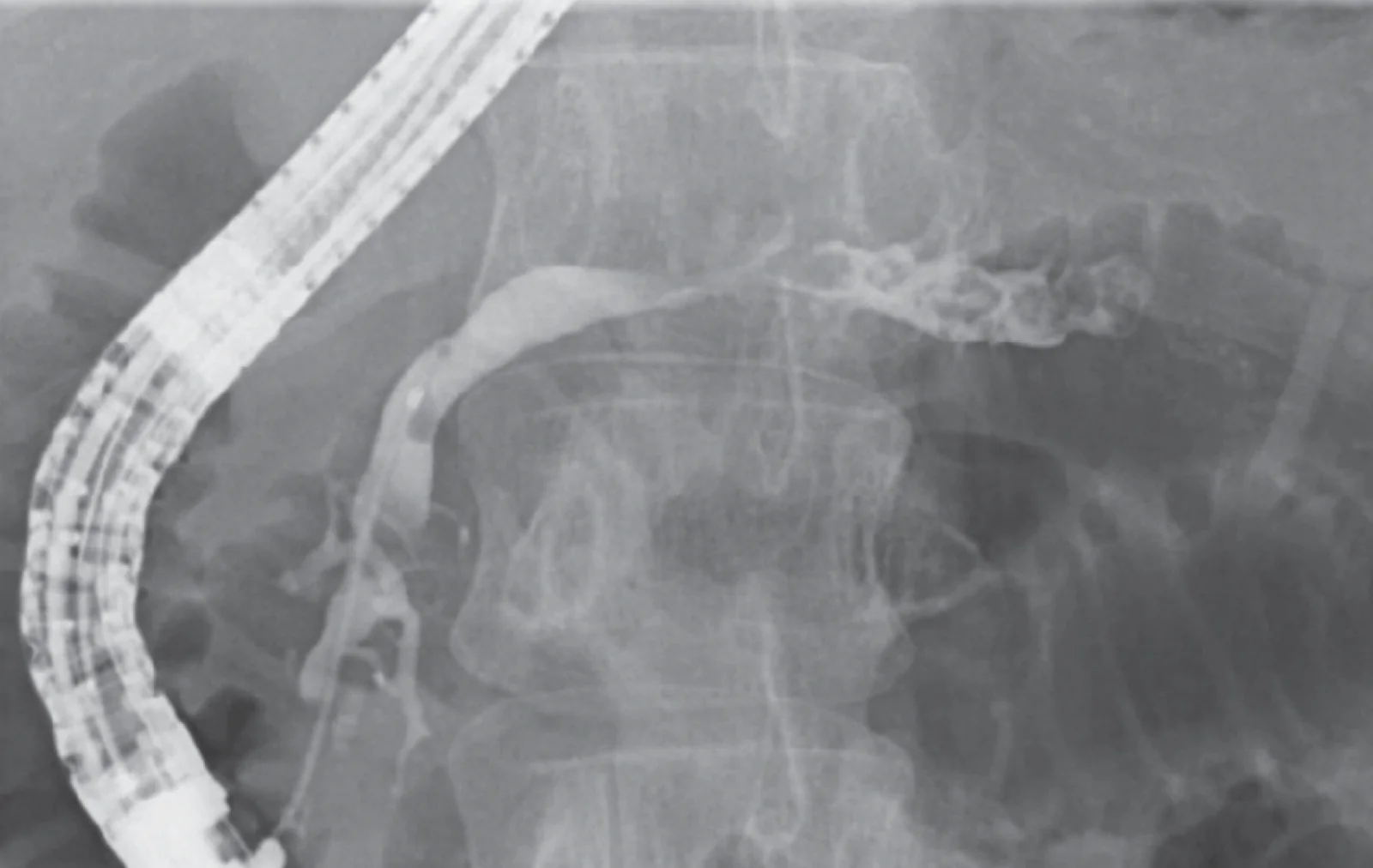
A pancreatogram showing a stricture in the pancreatic body and multiple upstream stones.

**Fig. 2:**
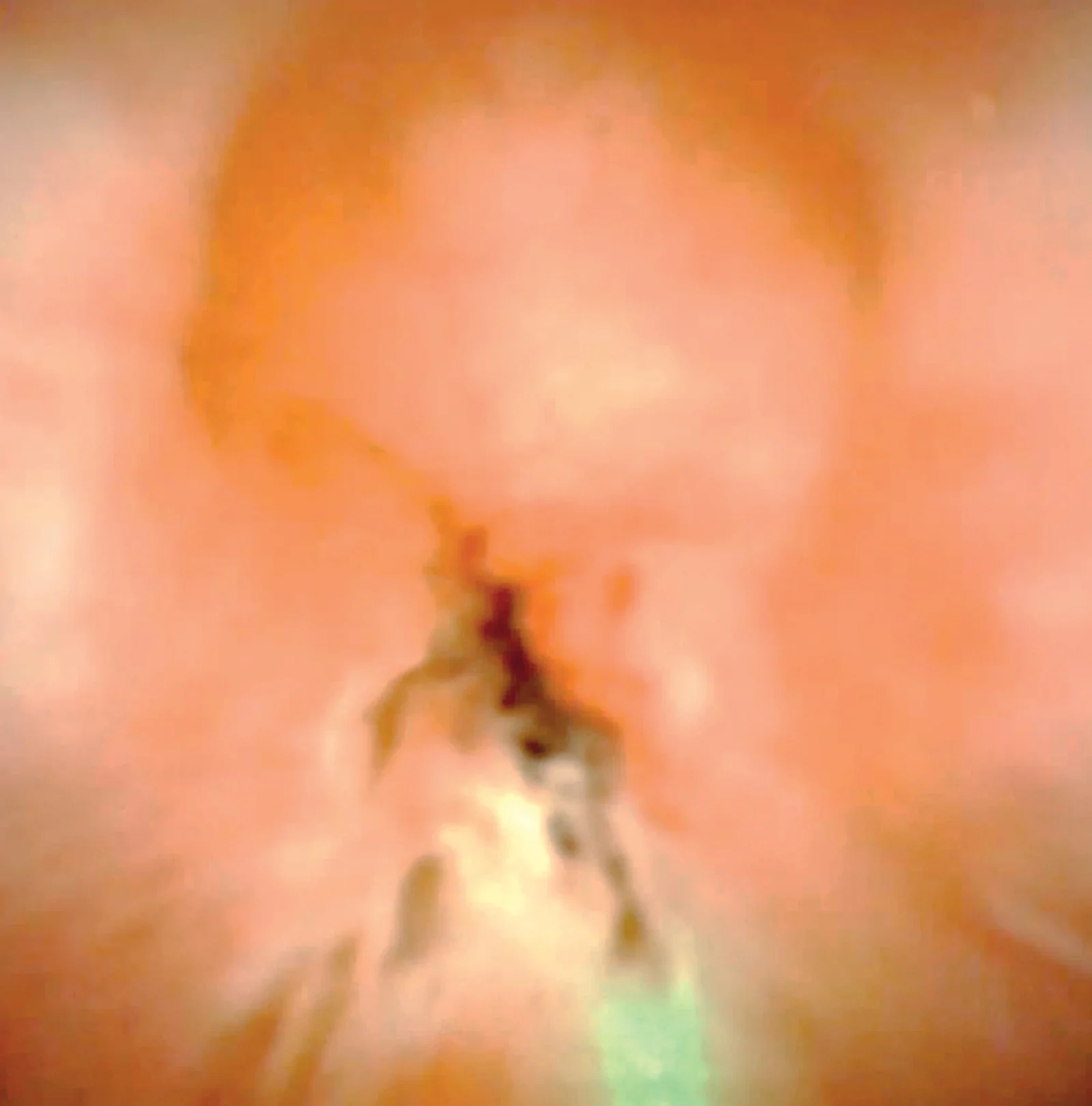
Peroral pancreatoscopy showing a severe pancreatic body stricture with band-like fibrosis. Pancreatoscopy-guided laser stricturoplasty was performed.

**Fig. 3:**
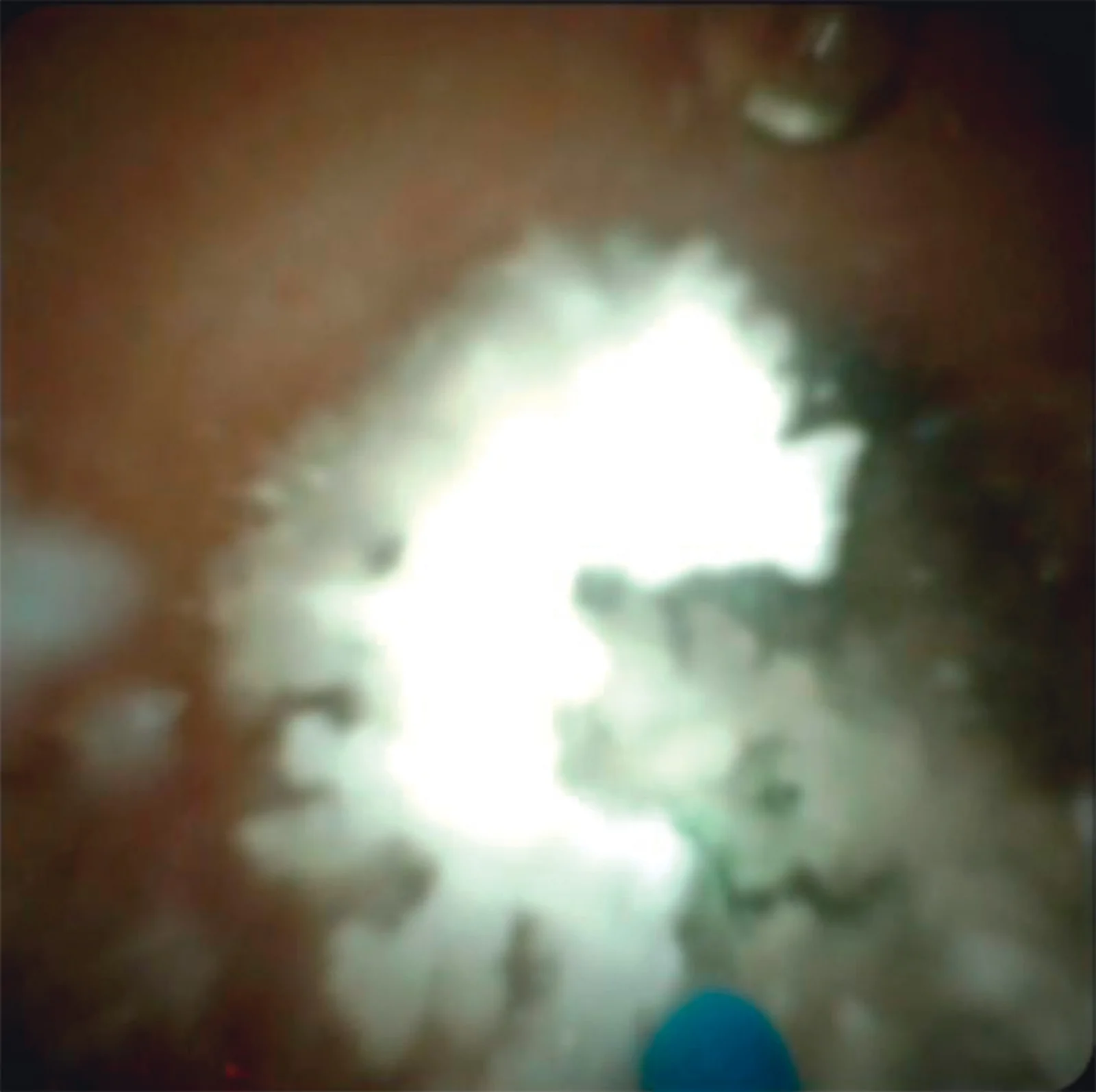
After pancreatoscopy-guided laser stricturoplasty, access to the pancreatic stones was achieved for laser lithotripsy.

**Fig. 4:**
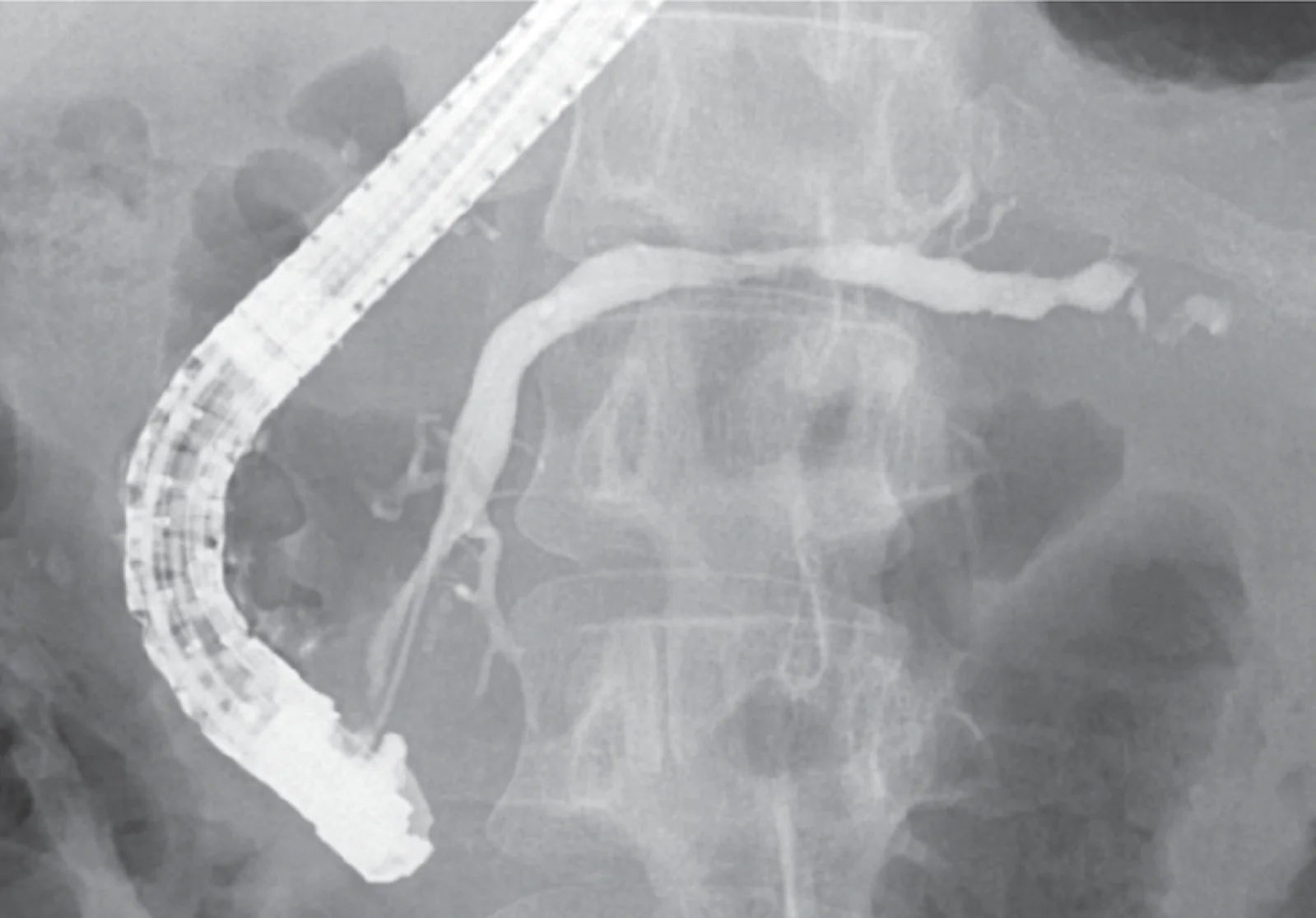
A final pancreatogram showing improvement of the stricture and complete stone clearance.

Endoscopy_UCTN_Code_TTT_1AR_2AH

## Declaration of GenAI Use

During the preparation of this manuscript, the authors used ChatGPT (OpenAI) solely for English-language editing to improve grammar, clarity, and readability. The authors reviewed and edited all artificial intelligence-assisted contents and take full responsibility for the final content of the manuscript.
